# Dynamic 3D Modeling for Human Sperm Motility through the Female Cervical Canal and Uterine Cavity to Predict Sperm Chance of Reaching the Oocyte

**DOI:** 10.3390/cells12010203

**Published:** 2023-01-03

**Authors:** Mayssam Nassir, Mattan Levi, Natan T. Shaked

**Affiliations:** Department of Biomedical Engineering, Faculty of Engineering, Tel Aviv University, Tel Aviv 69978, Israel

**Keywords:** uterus, vaginal delivery, 3D reconstruction, measurements of mechanical properties, finite element model, biomechanical simulation

## Abstract

Sperm motility in the female genital tract is a key factor in the natural selection of competent cells that will produce a healthy offspring. We created a dynamic three-dimensional (3D) mechanical model of human sperm cells swimming inside cervical canal and uterine cavity dynamic 3D models, all generated based on experimental studies. Using these simulations, we described the sperm cells’ behaviors during swimming inside the 3D tract model as a function of 3D displacement and time. We evaluated normal- and abnormal-morphology sperm cells according to their chances of reaching the oocyte site. As expected, we verified that the number of normal sperm cells that succeeded in reaching the fallopian tube sites is greater than the number of abnormal sperm cells. However, interestingly, after inspecting various abnormal sperm cells, we found out that their scores changed compared to swimming in an infinite medium, as is the case with in vitro fertilization. Thus, the interactions of abnormal sperm cells and the complicated geometry and dynamics of the uterus are significant factors in the filtering of abnormal sperm cells until they reach the oocyte site. Our study provides an advanced tool for sperm analysis and selection criteria for fertility treatments.

## 1. Introduction

The interaction of human sperm cells with the female reproductive system has a significant impact on fertilization. The ejaculated sperm cells are deposited in the female vagina, cervix, or uterus and travel inside the female reproductive system to fertilization sites [1]. On average, a few dozen to hundreds of sperm cells reach a fertilization site, where only a single sperm cell fertilizes an oocyte. This may be one of the strictest selection processes created by evolution, but its design and role are not fully understood. The correlation between sperm morphology, motility, and uterine interaction should affect the diagnosis and prognosis of male fertility, especially in the pathological cases of abnormal sperm morphology and motility. In addition, such analysis may help obtain a more informed sperm selection for in vitro fertilization (IVF), where a male sperm cell fertilizes a female oocyte in a dish. Because the physiological mechanisms and obstacles to sperm cells’ natural selection in the female body are bypassed in IVF, it is not possible to predict which individual sperm cell would be the one that is most likely to fertilize the oocyte naturally and result in a healthy child, especially in pathological cases. Moreover, the relationship between the morphology, motility, cell–surface interaction, and fertilization potential is still not completely clear, and, thus, various mathematical models have been proposed (e.g., [2]). Understanding sperm 3D motility while swimming inside the female reproductive tract may lead to improved sperm cell classification and sperm selection for IVF.

Sperm swims through the cervix and uterus to the oviduct, where it can fertilize the oocyte. The human cervix is composed predominantly of collagen and ground substance and contains relatively few smooth muscle cells [2,3,4]. The muscle fiber architecture of the cervix likewise consists of bundles running in an approximately circular fashion, two fiber systems running at acute angles, and the continuation of the fibers of the parametrium [5]. The inner and outer layers of the matrix have fibers, which are aligned longitudinally along the axis of the cervical canal [6]. The cervix is filled with cervical mucus, which is considered a major barrier to sperm, especially for the abnormal ones [7]. The cervix structure and the cervical mucus affect sperm motility toward the uterine cavity [8]. Previous microfluidic studies have shown that sperm cells are dynamically affected by the cervix folds and the cervical mucus flow [9]. The sperm cell crosses the cervical canal into the uterine cavity, which contains two dense families of muscle and collagen fibers that are mainly oriented in the circumferential direction [10]. Sperm cells swim through the uterus by their internal flagellar beating and with the aid of uterine contractions in the direction of the oviduct [11]. Uterine contraction characteristics, such as intensity, frequency, and duration, vary during the various phases of the menstrual cycle. In previous studies, the transabdominal ultrasound technique was used to study uterine activity in a noninvasive manner to evaluate the contraction parameters [12]. The inner structure of the human uterus is a physiological barrier that selects normal sperm cells [13,14,15]. Ultrastructural cell studies measured the influence of different morphological abnormalities on the ability of sperm to penetrate the cervical mucus and reach the oocyte [16]. They observed an incidence of sperm with abnormal heads after swimming in the cervical canal [17], whereas, in contrast, other studies reported that the cervical canal excluded abnormal sperm cells with defective heads [15,18]. The ability to evaluate sperm cells according to their morphology and swimming behavior in the female tract could be of great value for semen analyses, as well as a tool for predicting the types of spermatozoa that might be expected to gain access to fertilization sites.

In the current study, we developed a dynamic 3D mechanical model of sperm cell swimming inside the human female cervical canal and uterine cavity and scored the pathological cells according to their chances of reaching the oocyte site. We used 3D geometrical models of normal and abnormal sperm cells. The sperm cell models, including the full 3D sperm geometry, were constructed based on our previous experimental rapid optical acquisition method for individual human sperm cells during 3D movement [19,20]. The pathological sperm models include typical frequent defects in the sperm’s head and flagellum morphologies, which are based on real-world sperm pathologies according to the WHO 2021 guidelines [21]. In the current paper, we added a 3D mechanical model of the human female cervical canal and uterine cavity, which is based on human uterus ultrasound and magnetic resonance imaging (MRI) results [22,23,24,25,26] and tested the dynamic behaviors and interactions between the dynamic 3D female model and our dynamic 3D sperm models.

## 2. Methods and Materials

### 2.1. 3D Model of Human Female Cervical Canal and Uterine Cavity

Using 3D numerical simulation tools and previous ultrasound and MRI results, we built a geometrical human female cervical canal and uterine cavity. The dimensional parameters of the 3D geometrical model, such as uterine cavity volume, cervical canal length and diameter, and fundus distance, were taken from previous ultrasound and MRI measurements [22,23,24,25,26].

**Figure 1:**
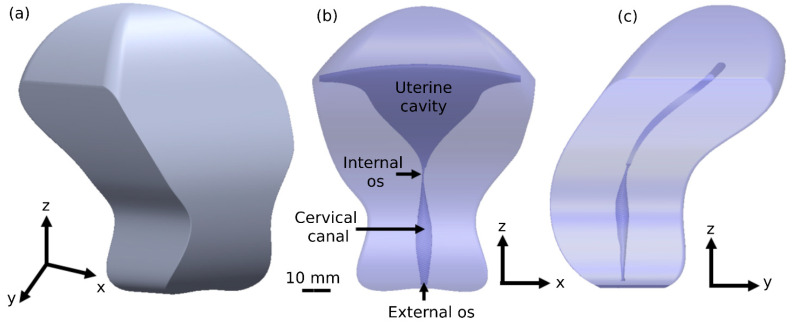
Female canal model and uterine cavity model. (**a**) 3D female tract geometrical model. Projections of the 3D geometrical model in the ‘x–z’ plane (**b**) and ‘y–z’ plane (**c**).

**Figure 2:**
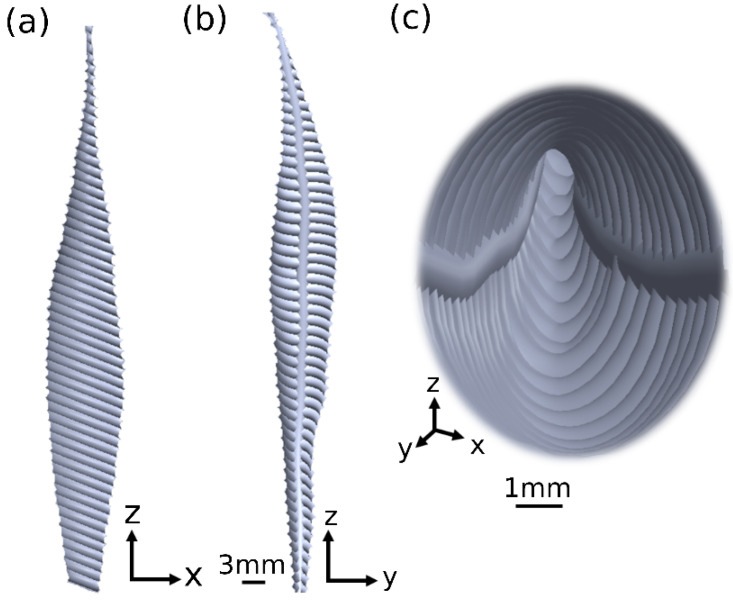
3D cervical canal model. (**a**,**b**) Side views of the cervical canal model containing the asymmetrical folds. (**c**) Inside view of the cervical canal model.

The 3D geometrical female model contains a connected tube with a pear-shaped geometry. The model possesses two parts of the female reproductive system, including the cervical canal and the uterine cavity (Figure 1). The cervical canal is protruded by two-sided longitudinal folds, which are mathematically described by trigonometric functions, altering the compartment radius (Figure 2). The cervical canal is approximated by a short tube, 35 mm long and 2.5 mm wide, with a radial symmetrical tapering reaching the two tips of the tube, approximated at 1.5 mm. The dimensional values of the cervical canal are fully described in Table S1. The internal layer of the cervical canal is composed of elliptical primary and secondary longitudinal folds, presenting the longitudinal endometrial folds. The full mathematical equations of the elliptical fold of the cervical canal are described in Table S2. The end of the cervical canal, or the internal os, is connected to the uterine cavity. Table S2 mathematically describes the uterine cavity as having a triangular shape and a mere slit-flattened anteroposterior. The inner surface of the 3D model is an elastic layer with a thickness of 10 mm, which describes the muscle layer of the uterus, or the myometrium [27,28]. The tips of the uterine cavity in the top portion are closed. The model was built in SolidWorks (Premium 2022, x64 Edition, Waltham, MA, USA).

### 2.2. Uterine Peristalsis

**Figure 3:**
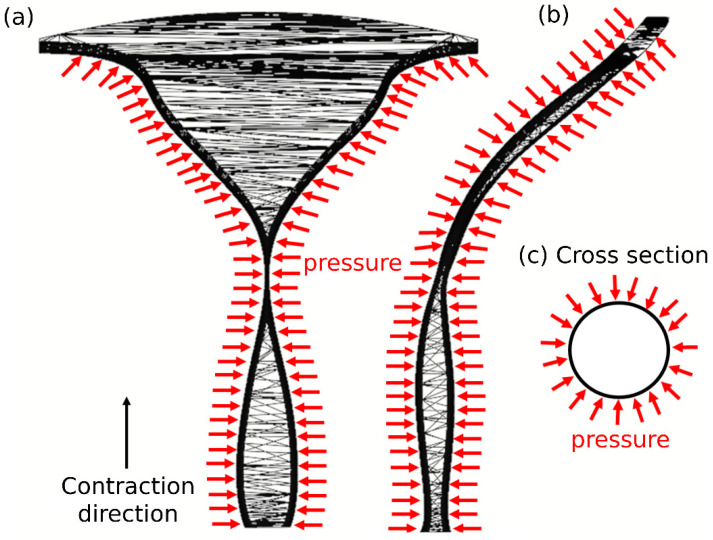
Pressure distributions along the 3D tract. (**a**,**b**) Side views of the direction of the pressure distribution. (**c**) Pressure distribution around a cross section of the 3D tract model.

Uterine contractions occur through the muscle layer of the uterus during the menstrual cycle. Here, we applied uniformly distributed pressure on the muscle layer as a function of distance and time (Figure 3). Distributed pressure was applied vertically around each cross section of the 3D tract model as a function of time (see Figure 3c). For each contraction, the pressure was applied in the cross section of the external os opening and propagated along the 3D genital model until reaching the fallopian tube sites. We used uterine contraction characteristics reported in earlier studies, which included contraction intensity (20 mmHg), frequency (1.5 1/min), and duration (20 s) [12,29,30,31]. We assumed that the muscle layer is an isotropic compressible material and used a neo-Hookean constitutive model with a strain energy function as follows:

(1) $$W=\frac{\mu }{2}\left(I_{1}-3\right)+\mu ln\left(J\right)+\frac{\lambda }{2}ln^{2}\left(J\right),$$

(2) $$\;\lambda =\frac{\nu E}{\left(1+\nu \right)\left(1-2\nu \right)},$$

(3) $$\mu =\frac{E}{2\left(1+\nu \right)},$$

where $I_{1}$ is the first invariant of the right Cauchy–Green deformation tensor and J is the determinant of the deformation gradient tensor. The parameters μ and λ are the Lamé parameters that relate to the Young’s moduli (E=0.7 MPa) and the Poisson’s ratios (ν=0.49) of the muscle layer [6,32].

Video S1 shows 20 s of a single-wave uterine contraction that starts in the external os opening of the cervical canal and ends in the fallopian tube sites.

### 2.3. 3D Reconstruction of the Human Sperm Models

We used the 3D geometrical models of normal and abnormal sperm cells developed in our previous study [19] and tested them only for swimming in an infinite medium. In this previous study, we built a numerical mechanical sperm model based on ultra-rapid dynamic 3D imaging experimental results of sperm cells obtained by our group [20]. A normal-morphology sperm cell swimming in a fluid was acquired with our dynamic interferometric computer tomography method at a spatial resolution of 0.5 microns, achieving both the retrieval of the 3D refractive index profile of the sperm head and the detailed 4D localization of the thin, highly dynamic sperm flagellum. The acquisition duration was 1 s. Based on these experimental results, in [19], the dynamic geometrical sperm model was built, and we could then predict the swimming patterns for hours. In this previous work, we tested sperm cells that were only swimming in an infinite medium, which does not necessarily predict how sperm would behave in a woman’s body.

**Figure 4:**
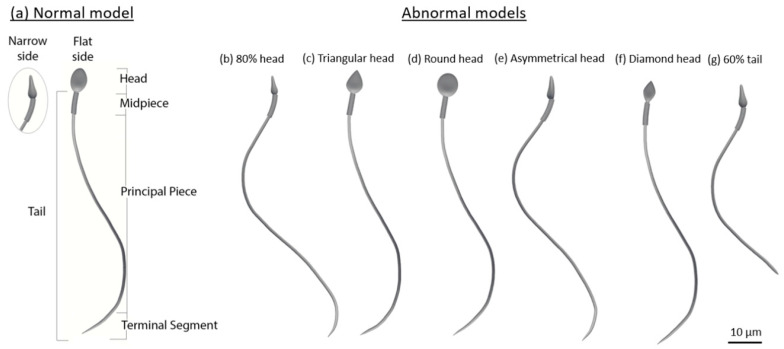
Normal and abnormal sperm cell models (according to the WHO classification [21]). (**a**) Normal sperm cell model. (**b**–**g**) Different typical morphological abnormalities among the sperm cells.

Our sperm models possess full sperm geometry with all internal organelles (a head, a flagellum, and a midpiece). The sperm head model contains a nucleus, an acrosome, and a membrane. The sperm midpiece (neck) model is composed of a centrosome and mitochondria, covered by a membrane. The sperm flagellum (tail) is constructed of nine doublet microtubules, a central double microtubule, a radial spoke triplet, nexin links, and dynein motors, which are covered by a membrane. In the current paper, we built the following seven detailed 3D dynamic sperm cell models: one for a normal sperm cell and six for sperm cells with abnormal morphologies and various typical abnormalities. These include defects in the morphology of the tail (a short tail), head size (a small head), and head shape (triangular, round, asymmetrical, and diamond heads) (see Figure 4).

### 2.4. Beat Pattern

The developed model is a mechanical–mathematical model based on kinetic laws and finite element motion in 3D. Each element of the model is described as a node in a mesh and is treated as an independent body based on the finite element theory. Forces were applied to the elements, each of which moved in the 3D space, creating its own 3D path. The composition of all elements described the path of the cell. Each dynein component was designated to be an element, sliding based on its curvature and position change. The inputs of our model are forces applied to the dynein components based on the “switch-point” principle, whereas the fixed forces describe the external influential forces, such as hydrodynamics and gradients. Specifically, we describe hydrodynamics as a fixed shear force that limits the elements’ movements along the progress axis. Using Frenet equations, we converted the applied forces to position and rotation changes for each element. Finally, we used the location and rotation changes to create a simulation of the beating patterns of sperm cells. For this purpose, the 3D axoneme sub-elements are divided into two groups of three or six filaments, which are located on opposing sides [33]. The axonemal beating is created by activating the dynein motors in the two filament groups intermittently based on the “switch-point” principle [33,34,35,36]. This principle specifies that the flagellar beat pattern is caused by the periodic switching of spatially restricted, asymmetrical dynein activation in the two filament groups. In other words, the axonemal bending in one direction is generated by activating the dynein motors in the same direction, while the dynein motors located along the second filament group on the opposite side are passive. Therefore, the action of the dynein motors at opposite sides of the axoneme is superimposed in an antagonistic manner by switching flagellar sides relative to the bending direction. The number of active dynein motors along each filament was determined to be *N* = 100 nodes. The beating patterns of the different models were unaffected by larger numbers of nodes, but they influenced the simulation runtime and memory. Low simulation resolutions were obtained with fewer than 100 nodes of dynein motors.

We have previously shown the 3D beating patterns of normal and abnormal models in a fluid by simulating the dynein sliding forces based on the “switch-point” principle [5]. Here, the normal and abnormal sperm models were considered to be hyperactivated sperm models. The 3D hyperactivated beating patterns of the different models were created by increasing the dynein sliding forces applied to the sperm models from a previous study [19]. The movement characteristics of the hyperactivation mode are represented by the flagellar amplitude, flagellar beat frequency, and straight-line velocity of the sperm models [37,38,39] (see Video S2).

### 2.5. Computerized Fluid Dynamic

Here, we study sperm swimming inside a viscous medium in the 3D female cervical canal and uterine cavity model. This viscous medium is considered to be a fluid that has the same thermophysical properties as the cervical mucus to imitate the biophysical environment system of the human cervical canal (Table S3, Figure S1). We solved Navier–Stokes equations to describe the flow of the cervical mucus in the 3D female cervical canal and uterine cavity model. To represent the Navier–Stokes equations, we used computerized fluid dynamic (CFD) analysis software by SolidWorks flow simulation. The following equations are described by formulations of mass, momentum, and energy conservation laws [40]:

(4) $$\frac{\partial \rho }{\partial t}+\frac{\partial \left(\rho u_{i}\right)}{\partial x_{i}}=0\;,$$

(5) $$\frac{\partial \left(\rho u_{i}\right)}{\partial t}+\frac{\partial }{\partial x_{j}}\left(\rho u_{i}u_{j}\right)+\frac{\partial P}{\partial x_{i}}=\frac{\partial }{\partial x_{j}}\left(\tau_{ij}+\tau_{ij}^{R}\right)+S_{i}\;,\;$$

(6) $$\frac{\partial \rho E}{\partial t}+\frac{\partial \rho u_{i}\left(E+\frac{P}{\rho }\right)}{\partial x_{i}}=\frac{\partial }{\partial x_{i}}\left(u_{j}\left(\tau_{ij}+\tau_{ij}^{R}\right)+q_{i}\right)-\tau_{ij}^{R}\frac{\partial u_{i}}{\partial x_{j}}+\rho \varepsilon +S_{i}u_{i}\;,$$

(7) $$E=e+\frac{u^{2}}{2}\;,\;\tau_{ij}=µS_{ij}\;,\;\tau_{ij}^{R}={µ}_{t}S_{ij}-\frac{2}{3}\rho \kappa \delta_{ij}\;,\;S_{ij}=\frac{\partial u_{i}}{\partial x_{j}}+\frac{\partial u_{j}}{\partial x_{i}}-\frac{2}{3}\delta_{ij}\frac{\partial u_{k}}{\partial x_{k}},$$

where ρ is the fluid density, t is the time, u is the velocity, P is the pressure, τ is the stress tensor, q is the heat flux, ε−κ is the turbulent model, µ is the fluid viscosity, $S_{ij}$ is the volume constant deformation rate, E is the total energy, and e is the internal energy.

### 2.6. Dynamic Simulations

To perform dynamic simulations on the 3D geometrical sperm models, we used a generation and processing toolbox in SolidWorks to create a 3D tetrahedral element mesh. The mesh densities of the different models vary between the cells. This variability can affect the simulation runtime but does not change the outcome measures (less than 3%). The models were assumed to be hyperelastic, uncompressible materials with the neo-Hookean constitutive model. The models were assumed to be uncompressible materials with uniform mechanical properties for all components, such as density. Since the cell head is the major component by weight, changing the density of the components is not expected to significantly affect the swimming behavior [19].

We applied different dynamic simulations to analyze the beating patterns of the seven sperm models swimming inside a viscous fluid in the 3D cervical and uterine cavity model. For each simulation, 100 sperm models with the same morphology were placed in the external os, which is the lower end of the cervical canal (see Figure 1b). Each sperm model was assigned a random initial position within the external os and was free to swim through the 3D cervical canal and uterine cavity model. The dynamic simulations were performed using SolidWorks 2022, Blender 2.91, and Python 3.3 SW (3D modeling and rendering package).

### 2.7. Outcome Measurements

1. The 3D swimming trajectories of the different models were analyzed for position maps, calculated by SolidWorks 2022 as mentioned above. The localized displacements that developed in the sperm head centroid of the normal and pathological models were determined as a function of time. The outcome measures included the following:
2. The 3D swimming trajectories of the sperm head centroid of the normal and pathological sperm models across the 3D human female cervical canal and uterine cavity model over 5 h. To ensure that an adequate sample of semen is available and contains the healthiest sperm with the highest chances for success, the recommendation is to perform IVF no later than one and a half hours from ejaculation. We decided to extend the simulation to five hours to make sure we covered a larger dynamic range over time.
3. The number of normal and pathological sperm models passing through the cervical canal and reaching the fallopian tube site.
4. The positions of normal and pathological sperm models inside the 3D cervical and uterine cavity model as a function of swimming time.
5. Scoring the various sperm cell pathologies according to their estimated success rates of reaching the oocyte and comparing them with the scoring results of the infinite medium sperm simulation performed in [19].

## 3. Results

### 3.1. 3D Swimming Trajectories of the Sperm Models through the 3D Tract Model

**Figure 5:**
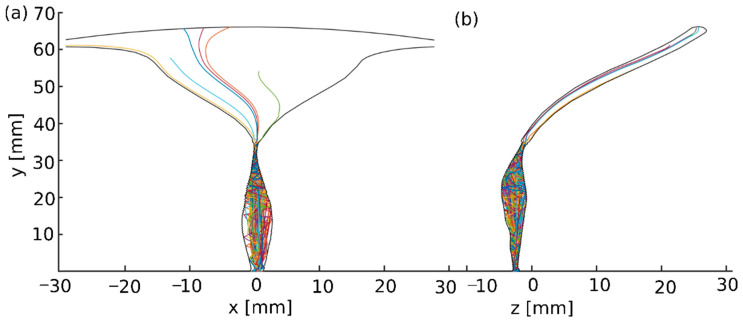
Swimming patterns of the normal sperm cell models in the human female cervical canal and uterine cavity. Two-dimensional trajectories in the anterior view (**a**) and the sagittal view (**b**). Each line color indicates a single sperm model path.

The 3D trajectories of the sperm head centroid of each sperm model across the 3D cervical canal and uterine cavity model were dynamically tracked. Figure 5 shows the two-dimensional trajectories of the normal sperm models through the tract model. We observed that the 3D paths of the normal sperm models indicate that the sperm head moved forward along the cervical canal with a small lateral displacement (Video S3). This swimming behavior is equivalent to the swimming pattern of normal sperm cells, as reported in previous experimental imaging studies tracking normal human sperm cells [19,20,21,41,42,43]. A total of 27 out of 100 of the normal sperm models succeeded in passing through the internal os, or the opening into the uterine cavity, and continued to swim inside the uterine cavity. At the internal os, most of the sperm models accumulated due to the narrow opening (which was approximately 0.6 mm side-to-side). Some of the normal models kept swimming near the wall of the uterine cavity and reached the fallopian tube sites, while other sperm models continued to swim straight inside the uterine cavity and accumulated in the fundus, which is the top part of the uterus (Figure 5).

During the swimming, we observed that the pathological sperm models displayed large variations in their 3D swimming behaviors through the cervical canal and the uterine cavity (Figures S2–S7). Compared to the normal models, the pathological sperm models exhibited different 3D swimming patterns, which showed significant 3D lateral displacements and twisted trajectories (Figures S2–S7).

The 3D swimming behaviors of the normal and abnormal sperm models near the surface of the cervical canal were significantly different. Video S4 shows a specific section of the different sperm models swimming near the longitudinal folds of the cervical canal. We observed that the different sperm models displayed various 3D swimming patterns when they swam near the cervical canal surface. The swimming section shown in Video S4 only describes a local path of the overall swimming behavior for each sperm model. The 3D swimming patterns were affected by the collision angle with the surface, collision velocity, cell-to-cell interactions, and the local geometry of the collision site.

### 3.2. Scoring of Sperm Pathologies Based on Their Positions in the 3D Tract Model

We calculated the percentages of the normal and pathological sperm models that passed through the cervical canal, in addition to the percentages of those that reached the fallopian tube sites (Table 1). The percentage of the normal sperm models that passed through the cervical canal and continued into the uterine cavity is the highest (27%). In addition, the percentage of the normal sperm models that reached the fallopian tube sites is 12%, which is higher than the percentage of the pathological sperm models. The quantities of the pathological sperm models that succeeded in entering the uterine cavity were lower than those of the normal sperm models (6–15%). Most of the sperm models accumulated in the internal os of the cervical canal due to its narrow opening. A few of the pathological sperm models reached the fallopian tube sites (0–4%) due to their 3D lateral displacement and twisted trajectories in the uterine cavity. These findings are equivalent to the sperm sample classifications inside the uterus according to their morphologies [16,17].

The percentages of the pathological sperm models with diamond (17%), small (15%), and triangular (11%) heads that succeeded in crossing the cervical canal were higher than those of the other pathological sperm models. The morphologies of these pathological sperm models are the most similar to those of normal-morphology sperm models when compared with the other pathological sperm models. Furthermore, the heads of these pathological sperm models are slightly smaller than the ones of normal sperm cells, which helps them pass through the internal os opening if they succeed in reaching it. Thus, high percentages of these models passed through the cervical canal and continued swimming through the narrow opening into the uterine cavity.

On the other hand, the numbers of pathological sperm models with asymmetrical heads (6%), round heads (4%), and short tails (6%), which succeeded in passing through the internal os opening, were lower than those of the other pathological models. A few sperm models with defective tails succeeded in reaching the internal os opening of the cervical canal. Furthermore, most of them accumulated in the internal wall of the cervical canal. For these sperm models, the tails did not have sufficient energy to move forward due to the fewer internal motors because of their short length, which weakened their normal beat. The pathological sperm models with round heads did not travel as far from their original location, which means that a small number of these cells crossed the cervical canal and reached the internal os opening. We can explain this by the load theory, according to which a large head increases the mass of the entire model and offloads the tail. In our case, the load is the total mass of the cell. Moreover, it will be difficult for these models to swim through the narrow diameter of the internal os opening and pass towards the uterine cavity, which explains the small number of these cells that passed through this barrier.

The 3D trajectories of the cell models with asymmetrical heads during swimming in the cervical canal were twisted and curved; therefore, only a few models were able to reach the internal os opening and continued swimming towards the uterine cavity. The asymmetrical heads caused asymmetric rowing inside the viscous fluid in the cervical canal, which caused these models to swim in twisted paths and get away from the internal os opening of the cervical canal.

### 3.3. Scoring the 3D Swimming Patterns of Normal Sperm Models as a Function of Time

**Figure 6:**
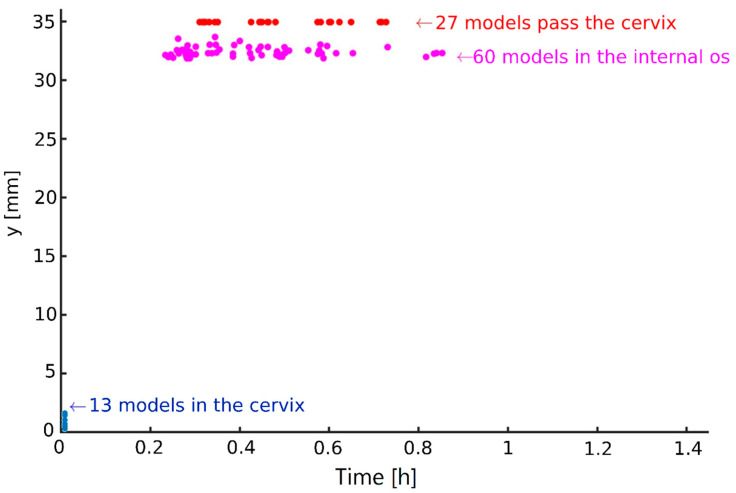
Normal sperm cell models’ behaviors in the cervical canal as a function of time. A total of 27 normal models passed through the cervical canal toward the uterine cavity in less than 0.7 h (red dots); 60 normal models were trapped in the internal os (purple dots); 13 normal models were trapped in the cervical canal (blue dots).

Here, we describe the positions of the normal and pathological sperm models inside the 3D cervical canal and uterine cavity model as a function of swimming time. The time that it takes the different sperm models to pass through the cervical canal, accumulate inside the cervical canal, or become trapped in the internal os opening was used to evaluate the sperm cell’s potential of reaching the oocyte. The runtime of the dynamic simulation was about 6 h of sperm models swimming inside the 3D cervical canal and uterine cavity model. After 5 h, the different sperm models did not change their positions and remained in the same local locations. The time that it takes the normal sperm models to pass through the cervical canal and continue swimming toward the uterine cavity was between 18 and 40 min (Figure 6). In less than an hour, most of the normal sperm models (60%) accumulated in the internal os opening of the cervical canal and were unable to continue swimming into the uterine cavity. A few of the normal models (13%) stopped swimming after a few seconds and accumulated inside the cervical canal near the external os opening. The normal sperm models that accumulated in the external or internal os openings became stuck in the cervical canal surface and were unable to free themselves to continue swimming, even after an hour of simulation runtime.

### 3.4. Scoring of Sperm Pathologies Based on Their Swimming Time in the 3D Tract Model

The swimming time of the normal sperm models through the 3D cervical canal and uterine cavity model was the shortest compared with the time of the pathological sperm models (Table 2). The normal sperm models were the fastest ones to cross the cervical canal, pass through the os internal opening, and reach the fallopian tube sites.

The sperm models with diamond or small heads swam faster than the other pathological sperm models. As we mentioned before, the morphologies of these pathological models are slightly different in size and shape from the normal-morphology sperm cells, which can explain their fast-swimming trajectories inside the 3D cervical canal and uterine cavity. On the other hand, it takes more time for the sperm models with triangular heads to pass through the cervical canal and reach the fallopian tube sites, despite their similarity in morphology with the normal cells. The sperm models with triangular heads swam forward with lateral displacements, which lengthened the swimming path and increased the swimming time to reach the fallopian tube sites. This can happen because of the asymmetry between the two tips of the triangular head, which causes asymmetric swimming inside the 3D cervical canal and uterine cavity model.

The swimming times of the sperm models with defective tails or rounded heads were longer compared with the other pathological sperm models. Sperm cells with defective tails have fewer internal motors in their shorter tails, which increases the total swimming time to reach the fallopian tube sites. Rounded heads increase the mass of the sperm and offload the tails; therefore, it took more time for the sperm models with rounded heads to cross the cervical canal and reach the fallopian tube sites. A few models with asymmetrical heads succeeded in passing through the cervical canal and continued swimming inside the uterine cavity, but none of them succeeded in reaching the fallopian tube sites. As we explained before, asymmetrical heads cause asymmetric swimming and large lateral displacements from the straight path. Therefore, these cells swim in long and twisted paths in the uterine cavity, far from the fallopian tube sites.

### 3.5. Scoring of Sperm Pathologies—Summary

**Figure 7:**
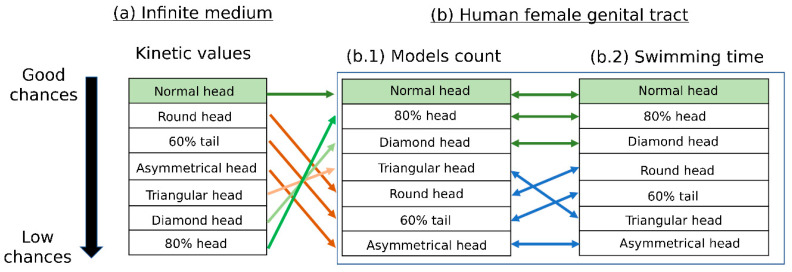
Scoring of sperm-pathology categories according to the cell chances of reaching the oocyte. Scoring of the various sperm cell pathologies based on the kinetic values of the infinite medium (**a**) [19], the model count (**b.1**), and the swimming time (**b.2**) results of the human female cervical canal and uterine cavity simulation. The arrows indicate changes in the scoring due to chances of reaching the oocyte.

We scored the various sperm pathologies according to their estimated success in reaching the oocyte based on the results described above. Figure 7 compares these results with the scoring results of the infinite medium simulation performed in our previous study [19]. As expected, the normal sperm cells have the best chance of reaching the oocyte, according to our simulation results in both the female body and the infinite medium. The number of normal sperm models that succeeded in passing through the cervical canal and reaching the fallopian tube sites is the largest compared to the pathological sperm models. The normal cell models were also the fastest. These results are equivalent to our previous report that calculated the kinetic parameters during swimming in an infinite medium to evaluate the sperm cells.

The sperm models with small or diamond heads had good chances of reaching the oocyte and the closest behavior to the normal ones due to their swimming time and model amounts that passed through the cervical canal and reached the fallopian tube sites. Thus, theoretically, they would reach the oocyte in second place after the normal cells. However, in our previous study, which described sperm swimming in an infinite medium, these pathological sperm models had a lower chance of reaching the oocyte. The sperm models with rounded heads, short tails, or asymmetrical heads swam slowly through the 3D cervical canal and uterine cavity. Furthermore, a few of these models succeeded in crossing the cervical canal and reaching the fallopian tube sites. Hence, these sperm models had lower chances of reaching the oocyte compared with the other pathological sperm models. Here, these results were also inconsistent with a previous study, which showed that these pathological sperm models had a high chance of reaching the oocyte.

Significantly, we found that the scoring of the pathological sperm models based on their swimming patterns in the 3D cervical canal and uterine cavity model is different from our previous scoring when swimming in a free infinite medium [19]. The interactions between the sperm cells and the female reproductive system largely affect the swimming behavior of the sperm cells, especially the abnormal cells; thus, the tools presented in the current paper, which also involve the female body, provide a more accurate evaluation of sperm cells.

## 4. Discussion

We described the swimming trajectories of different human sperm cell models and scored them according to their chances of reaching the oocyte by swimming inside a human female cervical canal and uterine cavity model. We developed a 3D dynamic simulation tool of a human female cervical canal and uterine cavity. The model geometry was constructed based on ultrasound and MRI results of a human uterus, which were performed in previous imaging studies [22,23,24,25,26]. In our model, we also applied uterine contractions (peristalses) using wave-like contractions along the tract model. The contraction characteristics were determined based on contraction recording results reported in earlier studies [12,29,30,31]. We used 3D geometrical models of normal and abnormal sperm cells to check their interactions with these female organs. The pathological sperm models include known frequent defects in the sperm head and flagellum morphologies, which are based on typical real-world sperm pathologies according to the WHO guidelines [21].

Full mechanical modeling of the 3D swimming patterns of sperm cells passing through the human female cervical canal and uterine cavity is an important factor in understanding sperm cell motility and behavior in the female reproductive system. Our advanced 3D mechanical model can improve sperm cell selection methods by understanding the swimming patterns of normal and abnormal sperm cells. We performed dynamic simulations for free swimming of the different sperm cell models inside the 3D dynamic model of the cervical canal and uterine cavity, evaluating the sperm cells by their chances of reaching farther and faster in the female body as a means for evaluating success in fertilization.

We observed that the normal sperm models swam forward along the cervical canal with a small lateral displacement and continued swimming straight inside the uterine cavity. In addition, we compared the normal sperm models with the abnormal sperm models, which showed different swimming paths in the tract model. The 3D trajectories of the pathological models have long and twisted paths, which ultimately prevent most of them from reaching the fallopian tube sites [19,20,21,41,42,43].

Most of the sperm models accumulated in the internal os opening of the cervical canal due to its narrow diameter. The narrow opening increases cell-to-cell and cell-to-surface collisions, which can affect the swimming paths due to changes in velocity, swimming directions, and rotational motions. Therefore, the swimming behavior of the sperm cells changed when they reached the internal os opening, and only a few cells succeeded in passing through this barrier and reaching the uterine cavity. This result matches the fact that this narrow passageway is considered a significant barrier against sperm cells [13,14,15]. We observed that some pathological sperm models became stuck in the inner wall of the cervical canal and were unable to release themselves to continue swimming forward. Sperm models with diamond, triangular, or asymmetric heads have sharp tips that can become stuck between the longitudinal folds in the cervical canal. The total mass of the pathological sperm models with rounded heads is large, which weakens the tail energy and decreases the total momentum; therefore, these models did not swim far in the cervical canal. Normal sperm models that succeeded in crossing the cervical canal and reaching the fallopian tube sites were the largest compared with the pathological models. The quantities of the pathological sperm models that reached the uterine cavity or the fallopian tube sites varied between the different types of abnormalities. Furthermore, we found that the normal sperm models moved the fastest in terms of 3D displacement inside the cervical canal and uterine cavity. The pathological models swam in longer trajectories inside the tract model for longer periods of time compared to the normal models. Our study confirmed the existence of selection for morphologically normal human spermatozoa within the cervical canal and uterine cavity, as previously and experimentally observed in sperm classification studies [16,17].

Lastly, we compared our findings with the scoring results of the 3D sperm cells swimming in an infinite medium, which were performed in our previous study [19]. Based on the different simulations, the normal sperm models swam in the most linear paths and had the highest chances of reaching the oocyte compared with the pathological ones. On the other hand, the scoring of the pathological sperm models according to their chances of reaching the oocyte changed when the models swam inside the 3D geometry model of the female reproductive system. Hence, besides the cell morphology influence, swimming under the female body environmental conditions significantly affects the behavior of sperm cells, especially the abnormal sperm cells. Moreover, both cell morphology influence and swimming in the complicated geometry of the uterus are significant factors for filtering pathological sperm cells until they reach the oocyte site. Therefore, the incorporation of environmental parameters, such as 3D real geometry, viscosity, and biomechanics, can promote an advanced study of sperm cell classification and evaluation.

Sperm density and total numbers may affect the sperm movement in space and can vary dramatically among patients. The limitations of our study include the fact that only 100 sperm models were placed in each simulation (per each sperm pathology). This is due to the great computational power required for such a dynamic, highly detailed 3D simulation of each swimming sperm cell. The sperm models were assumed to be uncompressible materials with uniform mechanical properties for all components, such as density. Since the cell head is the major component by weight, changing the density of the components is not expected to significantly affect the swimming behavior. Each simulation was performed at least ten times to increase its reliability, with repeated behavior observed. Simplifying the geometrical model of the sperm cell will optimize the simulation assumptions and conditions, and it is expected to allow for the inclusion of thousands of sperm cells within the female reproductive system.

Another limitation is the fact that only a part of the female reproductive system was included. Improvements to the 3D model of the genital tract by adding the two fallopian tubes can be a more accurate tool to describe the behavior of the sperm cells inside the female reproductive system.

In our future studies, we plan to increase the sperm cell numbers and test the cilia–cell interactions in the fallopian tubes’ geometry to better present the real situation in the entire female uterus.

To conclude, we have presented an advanced modeling tool for analyzing the sperm–female body interactions and scoring the 3D movements of healthy and pathological human sperm cells inside the cervical canal and uterine cavity of the human uterus. Our method is based on a multidisciplinary approach that combines prior knowledge of the sperm cells’ internal structure, a description of their flagellar beating patterns, and free swimming inside a 3D reconstructed cervical canal and uterine cavity models. This method may lead to changes in sperm cell classification and evaluation methods, as well as a biophysical analysis tool to fill in the gaps from previous studies. Specifically, understanding the migration of normal and pathological human sperm cells inside the female reproductive system can improve the strategies of human sperm cell selection and fertility evaluation. For example, this study implies that sperm cells with certain pathologies should be preferred to those with other pathologies (Figure 7). Future clinical studies are expected to validate our conclusions.

Our study opens the door to additional biophysical studies in evaluating sperm cell behavior and sheds light on the chances of a specific sperm cell reaching an egg naturally, which might lead to new standardizations in sperm grading and analysis.

## Figures and Tables

**Table 1:** Percentages of the normal and abnormal sperm models passing through the cervical canal (left column) and reaching the fallopian tube site (right column).

	Pass the Cervical Canal	Reach the Fallopian Tube Sites
**Normal models**	27%	12%
**Diamond head models**	17%	3%
**80% head models**	15%	4%
**Triangular head models**	11%	4%
**Asymmetrical head models**	6%	0%
**60% tail models**	6%	1%
**Round head models**	4%	2%

**Table 2:** Time positions (average) of the normal and abnormal models through the human female cervical canal and uterine cavity.

	Pass the Cervical Canal [h]	Reaching the Fallopian Tube Sites [h]
**Normal models**	0.44	1.28
**Diamond head models**	0.65	1.86
**80% head models**	0.8	1.95
**60% tail models**	0.9	2.5
**Triangular head models**	0.92	2.72
**Round head models**	1.9	3.35
**Asymmetrical head models**	2.17	Did not reach the fallopian tube site

## Data Availability

All data are available from the corresponding author upon a reasonable request.

## Supplementary Materials

The following supporting information can be downloaded at: https://www.mdpi.com/article/10.3390/cells12010203/s1, Table S1: Parameters used in implicit functions for description of the human female cervical canal; Table S2: The equations for the description of the cervical canal and the uterine cavity; Table S3: Thermo-physical properties of the cervical mucus; Figure S1: The viscosity of fresh human cervical mucus samples as a function of shear rate; Figure S2: Abnormal sperm models with short tail swimming patterns in the human female cervical canal and uterine cavity model; Figure S3: Abnormal sperm models with small head swimming patterns in the human female cervical canal and uterine cavity model; Figure S4: Abnormal sperm models with small head swimming patterns in the human female cervical canal and uterine cavity model; Figure S5: Abnormal sperm models with diamond head swimming patterns in the human female cervical canal and uterine cavity model; Figure S6: Abnormal sperm models with triangular head swimming patterns in the human female cervical canal and uterine cavity model; Figure S7: Abnormal sperm models with asymmetrical head swimming patterns in the human female cervical canal and uterine cavity model; Video S1: Uterine contractions; Video S2: Normal and hyperactivated sperm cells free swimming; Video S3: Normal sperm cells swimming through the cervix; Video S4: 3D Sperm cells swimming near the cervix surface. References [44,45] are cited in the supplementary materials.
